# Childhood maltreatment in adult offspring of Portuguese war veterans with and without PTSD

**DOI:** 10.3402/ejpt.v5.20198

**Published:** 2014-02-04

**Authors:** Aida Dias, Luisa Sales, Rui M. Cardoso, Rolf Kleber

**Affiliations:** 1 Faculty of Social Sciences, Utrecht University, Utrecht, The Netherlands; 2 Centro de Trauma—Ces, Coimbra, Portugal; 3 Medical Psychology Service, Faculty of Medicine, University of Porto, Porto, Portugal; 4 ArQ Research Foundation, Diemen, The Netherlands

**Keywords:** Abuse, neglect, war, PTSD, intergenerational transmission, offspring, Portugal

## Abstract

**Background:**

The colonial war that Portugal was involved in between 1961 and 1974 had a significant impact on veterans and their families. However, it is unclear what the consequences of this war are, in particular with regard to levels of childhood maltreatment (CM) in offspring.

**Objective:**

Our study aims to analyze the influences of fathers’ war exposure and posttraumatic stress disorder (PTSD) on the offspring's CM and simultaneously test the hypothesis of the intergenerational transmission of father–child CM.

**Method:**

Cross-sectional data were collected, using the Childhood Trauma Questionnaire—Short Form, from 203 adult children and 117 fathers. Subjects were distributed according to three conditions based on the father's war exposure status: did not participate in war, or non-war-exposed (NW); participated in war, or war-exposed (W); and war-exposed with PTSD diagnosis (WP). The data were examined using correlations, variance/covariance, and regression analyses.

**Results:**

Children of war veterans with PTSD reported more emotional and physical neglect, while their fathers reported increased emotional and physical abuse exposure during their own childhood. Significant father–child CM correlations were found in the war veteran group but less in the war veteran with PTSD group. Father CM predicted 16% of offspring CM of children of war veterans.

**Conclusions:**

The father's war-related PTSD might be a risk factor for offspring neglect but potentially a protective one for the father–child abuse transmission. War-exposed fathers *without* PTSD did transmit their own CM experiences more often. Therefore, father's war exposure and father's war PTSD may each be important variables to take into account in the study of intergenerational transmission of CM.

Portugal was involved in a colonial war between 1961 and 1974, during which all young adult men were obliged to perform military service. Those who escaped this duty did so by leaving the country; in specific cases, familial obligations could provide a ground for exemption from service. About 1 million men were deployed to the African continent during military service, most of whom experienced war for 24 months. For a country with about 10 million inhabitants, the number of soldiers directly exposed to the war is extremely high, underlining the need for better understanding of the consequences of this period for society.

Studies conducted among Portuguese war veterans indicated that 10.9% of war-exposed men developed posttraumatic stress disorder (PTSD) (Albuquerque, Soares, Jesus, & Alves, 2003), and that around 37% of veterans reported that they had showed violent behaviors at home after their return (Maia, MacIntyre, Pereira, & Fernandes, 2006). Besides being exposed to their fathers’ absence, children of Portuguese war veterans could have been exposed to impaired parenting skills caused by war exposure or PTSD. However, most children are unaware of the past war experience of their fathers and therefore lack knowledge about the possible consequences of these experiences (Sales, Dias, & Mota-Cardoso, 2011).

## Childhood maltreatment

Childhood maltreatment (CM) is a recognized risk factor for mental, social, and physical health problems (Edwards et al., 2005) and is considered to be highly prevalent in developing (Akmatov, 2011) and developed countries (Gilbert et al., 2009). According to Hussey, Chang, and Kotch (2006), characteristics of families with CM include poor parent mental health and abused or neglected caregivers.

A suggested cause for CM is intergenerational transmission (Egeland, 1993): parents exposed to CM have a greater probability of maltreating their own children. However, depending on different methodological approaches, research studies found rates for CM intergenerational transmission ranging between 7 and 70% (Egeland, 1993; Gil, 1970). A review of three longitudinal studies concluded that roughly 30% of CM rates could be explained by intergenerational transmission (Kaufman & Zigler, 1987). Nevertheless, a review by Thornberry, Knight, and Lovegrove (2012) concluded that studies with better methodologies did not provide enough support for this hypothesis. The same authors suggested that there is a need to include independent responders among analyzed samples, to target community subjects and not only at-risk populations, and to address other mediating factors in CM intergenerational transmission.

A study by Pears and Capaldi (2001) concluded that 23% of CM rates could be explained by intergenerational transmission, but the authors also found that parents with greater exposure to CM and with high levels of depression and PTSD were less abusive toward their children. Pears and Capaldi suggested that depressed parents who are experiencing PTSD may avoid interaction with their children, reducing the probability of perpetrating abusive forms of CM.

## Children of war veterans

War deployment is a period of adaptation for military families. Increased levels of stress can be caused by the deployed parent's absence from the family and by the recognition of increased risk for the deployed member that went to war. White, Burgh, Fear, and Iversen (2011) reviewed studies on the consequences for children whose fathers were deployed in wars in Iraq and Afghanistan. They concluded that these children revealed increased emotional and behavioral problems. Parental psychopathology was highlighted as one of the most prominent risk factors. Similar results were found in a study of CM in Holocaust families (Yehuda, Halligan, & Grossman, 2001). Results of Dekel and Goldblatt's (2008) study also corroborate the conclusion that parental PTSD is a risk factor for offspring psychopathology, while reviewing studies on intergenerational war trauma transmission. A recent review by Leen-Feldner et al. (2013) concluded that offspring of parents with PTSD have an increased risk for psychological problems. The authors suggested parenting behaviors as potential transmission mechanisms. CM follows within this framework: parents who were maltreated during childhood may have an increased risk for PTSD; at the same time, their parenting skills might be impaired, not only because of their PTSD symptoms but also because of the inadequate educative practices they have experienced (Muller, Hunter & Stollak, 1995).

Rentz et al. (2007) argued that, during the Gulf War, CM rates among the children of military families increased substantially (around 30%) compared with CM rates in the community as a whole. Familial stress caused by the deployment cycle was advanced as one of the probable causes. As suggested by White et al. (2011), parental psychopathology might be developed or aggravated as a consequence of war exposure in both parents—in the caretaker that assumes parenting duties during deployment or, after deployment, in the war veteran. A question that, to our knowledge, has been poorly researched is how parental war deployment relates to parent–child transmission of CM.

## CM in war veterans with PTSD

Although war-related PTSD is thought to be caused by the experience of combat and by exposure to war atrocities, it is also known that pre-deployment conditions may influence the risk for PTSD. Childhood adversity has been recognized as a relevant risk factor for PTSD (Brewin, Andrews, & Valentine, 2000) and more specifically for combat-related PTSD and depression (Berntsen et al., 2012). In the U.S. Marines, it was also verified that exposure to CM, namely physical neglect, was related to post-deployment PTSD (LeardMann, Smith, & Ryan, 2010). Lanius, Frewen, Vermetten, and Yehuda (2010) suggested that early life adversity can be an important factor in explaining the emotional dysregulation that characterizes PTSD. CM occurs in important developmental stages and involves unhealthy interactions with significant attachment figures; it can modify adaptive patterns of reaction to stressful events, making them less flexible and sometimes inefficient when facing potentially traumatic events.

## Research goals

Although more than 30 years have passed since the end of the Portuguese colonial war, its consequences remain poorly analyzed. Scientific literature on the consequences of fathers’ exposure to war and PTSD for their offspring raises relevant questions. To our knowledge, it is unclear whether offspring CM might be related to: (1) fathers’ exposure to war, (2) father's war-related PTSD, or (3) fathers’ pre-deployment characteristics, such as CM exposure. Our study investigated these questions among Portuguese families.

## Method

### Design

This study is a cross-sectional investigation using data collected between 2008 and 2010 within a larger project, “Children of Colonial War: Post Memory and Representations.” Data were collected with authorization from the Portuguese Commission for Data Protection (CNPD) and from the Ethical Commission of the Military Hospital of Coimbra. Data pertaining to sociodemographics, father's war exposure and war-related PTSD diagnosis, and CM were selected for analysis. CM data were collected from children and their fathers using the Childhood Trauma Questionnaire—Short Form (CTQ-SF; Bernstein et al., 2003).

### Participants

Families with fathers with a war-related PTSD diagnosis (WP) were recruited from the Department of Psychiatry of the Military Hospital of Coimbra. The diagnosis of PTSD due to war exposure was identified 10 or more years before publication of the law that made benefits available for Portuguese war veterans with PTSD. Thus, we assume that the PTSD diagnosis was not affected by the possibility of legal reparation. Diagnosis was established by clinical interview and follow-up, using DSM-III-R and DSM-IV clinical criteria, and was later confirmed by the Clinician-Administered PTSD Scale (CAPS). This group of war veterans had been deployed for 24 months in Africa. They had been exposed to combat and had reported the death of comrades or the killing of civilians as the most traumatic events they experienced. Their symptoms began during their deployment or immediately after returning from war. Subjects with a principal diagnosis of depression, psychotic disorders, and/or substance abuse disorders were not selected. Thirty-five war veterans with PTSD were invited to participate in the study, but four declined. One of these refused to be asked about war memories, and three cited the unavailability of their children to participate.

Recruitment of children of war-exposed (W) and non-war-exposed (NW) fathers was performed using the snowball technique beginning with personal contacts by the researchers. Portuguese adults born between 1960 and 1985 were recruited. These adults then invited their families to participate. Families were assigned to two groups, depending on the father's status—father participated in war and father did not participate in war. W fathers had been deployed in a combat war scenario for 24 months. Families were not included in the W group if the father received psychiatric treatment because of war-related problems, as assessed during the interview.

Altogether, data from 203 adult children and 117 fathers were included. Paired father–child dyads were divided into three groups, according to the father's status: W, when the father had participated in the war; WP, when the father had participated in the war and was diagnosed with war-related PTSD; and NW, if the father had not participated in the war. Demographics are described in Table 1.

**Table 1:** Sample demographics

	Children (*n*=203)			Fathers (*n*=117)		
		
Demographics	W (*n*=95)	WP (*n*=60)	NW (*n*=48)	W (*n*=56)	WP (*n*=31)	NW (*n*=30)
Age mean (years)	33	35	34	63	64	61
Gender						
Female	52	34	22			
Male	43	26	26			
Marital status						
Single	41	19	22			
Married/with partner	45	39	23	54	29	29
Separated/divorced/widow	6	2	3	2	1	0
Education						
4 years or less		1	1	21	18	8*
Between 5 and 9 years	8	10	2	9	4	4
Between 10 and 12 years	19	13	8	17	3	4
Higher education	66	36	37	9	6	14*
Employment status						
Paid job	89	53	43	41	20	22
Student	4	2	3			
Unemployed	1	3	1		2	1
Pensioner/retired		1		15	9	7

* *Note*: Table numbers represent the n for each demographic category, except for age. W, war exposed; WP, war exposed with PTSD; NW, non-war exposed. Chi-square tests were conducted for each demographic category in children and fathers. Only in father education were found significant differences, with NW group having more subjects with higher education

* less subjects with low education (*χ* ^2^(3) = 17.163 with *p*≤0.009).

### Instruments

A brief, structured interview was used to collect sociodemographic and war-related data. The Childhood Trauma Questionnaire—Short Form (CTQ-SF; Bernstein et al., 2003) was used to assess CM. CTQ-SF is a widely used, retrospective, self-reported questionnaire that assesses different types of CM, and it provides a general CM score. It contains 28 items describing specific maltreatment experiences. Items are classified into a five-point Likert scale according to the frequency of CM exposure until the age of 15 years. The CTQ-SF provides scores for five different types of CM—emotional abuse, sexual abuse, physical abuse, emotional neglect, and physical neglect. Internal consistency studies revealed a Cronbach's alpha varying between 0.61 (for physical neglect) and 0.95 for (sexual abuse) in clinical populations (Bernstein et al., 2003) and between 0.58 (for physical neglect) and 0.94 (for sexual abuse) (Scher, Stein, Asmundson, McCreary, & Forde, 2001) in community samples. Results on time stability, as well as convergent and divergent validity, were considered to be good (Bernstein et al., 2003; Pavio & Cramer, 2004). Dias et al. (2013) confirmed the original CTQ-SF factorial structure in a Portuguese community sample of 746 subjects. Cronbach's alpha varied from 0.47 for physical neglect, 0.71 for emotional abuse, 0.72 for sexual abuse, 0.78 for physical abuse, to 0.79 for emotional neglect. The overall scale has a Cronbach's alpha of 0.84.

### Data analyses

Comparison of sociodemographic data on groups was performed with chi-square tests for categorical variables, and Student's *t*-test for continuous variables. Differences for CM global score by group and sex were first analyzed through analysis of variance (ANOVA) to check the differences in the global CM score for children and fathers, followed by Tukey post-hoc tests. Separated multivariate analyses of variance (MANOVAs) were performed to analyze the CM subtype differences in fathers and children. CM father–child Spearman's correlation coefficients and regression analysis were conducted to inspect the associations of different types of CM. Multivariate analysis of covariance (MANCOVA) model was performed to analyze the differences in CM subtypes by group and gender, adjusting for confounding effects by five subtypes of father CM. R^2^ and partial eta squared was used to compare effect sizes.

## Results

Sociodemographic characteristics of groups were examined using the Student *t*-test for age, and the chi-square test for sex, education, marital status, and job status. Children′s groups were equivalent in all the analyzed characteristics. The groups of fathers were equivalent in terms of age, and marital and job status, but not in education. The NW group had more subjects with higher education and fewer subjects with 4 or less years of education [*χ* ^2^(3) = 17.163 with *p =* 0.009].

### CM in fathers

A one-way ANOVA was applied to analyze the differences in the father's CM (the CTQ-SF global score, or CTQS) across the three groups (*n =* 117). A significant difference was detected [*F*(2, 185) = 6.030, *p =* 0.003]. Tukey post-hoc test comparisons revealed that WP fathers self-reported higher levels for CM (*M =* 39.78, *SD =* 11.18) than W fathers (*M =* 35.85, *SD =* 8.67), with *p <* 0.038, and NW fathers (*M =* 33.47, *SD =* 7.94), with *p <* 0.003. W and NW fathers did not differ in terms of CM global scores (*p =* 0.323).

To analyze specific differences among CM subtypes in the fathers’ groups, one-way MANOVA was performed. A statistically significant effect was obtained, Pillai's trace = 0.162, *F*(10, 222) = 1.95, *p*=0.040. Group differences between W and WP fathers were observed for emotional abuse [*F*(2, 117) = 3.00, *p*=0.033, *M*_W_=6.23, *SD =* 2.42, and *M*_WP_=7.77, *SD =* 3.5] and for physical abuse [*F*(2, 117) = 3.845), *p*=0.024, *M*_W_=5.92, *SD*=2.45, and *M*_WP_=7.32, *SD =* 3.35]. Higher scores for emotional and physical abuse were found for WP fathers when compared with W fathers. No significant differences in emotional abuse were found between WP and NW fathers. W and NW fathers did not differ in any of the assessed CM subtypes.

### CM in offspring

CM differences in offspring were first analyzed using one-way between-subject ANOVA, comparing the CTQ-SF global score (CTQS) among the three groups of children. Results showed a significant effect of father's war status on CM [*F*(2, 200) = 6.433, *p*=0.002]. Post-hoc comparisons using Tukey's test indicated that the offspring CTQS mean score for the WP condition (*M =* 35.62, *SD =* 8.08) differed significantly from the W condition (*M*=31.83, *SD*=6.70), with *p*=0.004, and from the NW condition (*M*=31.44, *SD*=6.57), with *p*=0.007. No significant differences were found between the W and NW conditions. These results suggested that war PTSD in fathers does have an effect on offspring CM, although father's war exposure did not appear to significantly increase offspring CM.

Gender differences in offspring CM were analyzed with one-way MANOVA. A significant effect was detected, Pillai's trace = 0.099, *F*(5, 197) = 4.33, *p*=0.001, partial eta squared = 0.099. Differences were significant for emotional abuse (*p*=0.046), for physical abuse (*p*=0.040), and for sexual abuse (*p*=0.023). Greater scores for emotional abuse and sexual abuse were reported by females, and higher scores were reported by males for physical abuse (Table 2).

**Table 2:** Comparison of mean differences of adult self-reported offspring childhood maltreatment by father's war status and by gender (*n*=203)

	Children									
	
	Father's war status						Gender			
		
CTQ-SF subscales	W (*n*=95)		WP (*n*=60)		NW (*n*=48)		Male (*n*=108)		Female (*n*=95)	
	M	SD	M	SD	M	SD	M	SD	M	SD
Emotional neglect	8.71^a^	3.20	10.68a,b	3.85	8.29^b^	3.38	9.22	3.27	9.16	3.82
Emotional abuse	6.95	2.61	7.83	3.17	6.98	2.24	6.81^g^	2.25	7.57^g^	3.05
Physical neglect	5.44^c^	1.14	6.44c,d	2.03	5.71^d^	1.38	5.71	1.31	5.87	1.75
Physical abuse	5.60	1.63	5.51	1.32	5.42	0.96	5.74^h^	1.63	5.34^h^	1.13
Sexual abuse	5.14	.64	5.15	0.55	5.04	0.29	5.03^i^	.17	5.20^i^	.72
CTQ sum	31.84^e^	6.7	35.62e,f	8.08	31.43^f^	6.57	32.52	6.54	33.16	7.92

*Note*: W, war exposed; WP, war exposed with PTSD; NW, non-war exposed.

a–i Means sharing the same letter significantly differed from each other, with *p <* 0.05.

### Father–child CM relations

Spearman correlation coefficients were analyzed to study the associations between father and child CM scores (Table 3). The W group showed significant correlations in almost every CM subtype, excluding sexual abuse. The highest correlation was found between father's emotional abuse and child's emotional neglect (*r =* 0.41, *p*<0.001). CTQS father–child correlations in the W group registered a correlation of 0.42 (with *p*<0.001). A different correlation pattern was found in the WP group. Significant correlations were only found between father's physical neglect and child's physical abuse (*r*=0.34, *p*<0.009) and between father's sexual abuse and child's emotional neglect (*r*=0.26, *p*<0.048). In the NW group, no significant correlations were encountered.

**Table 3:** Spearman coefficient correlations between father–child childhood maltreatment

Groups	Fathers	Emotional abuse	Emotional neglect	Physical neglect	Physical abuse	Sexual abuse	CTQ sum
War *n*=91	Children						
	EA	0.32**	0.16	0.214*	0.30**	−0.10	0.25*
	EN	0.41**	0.33**	0.30**	0.24*	0.09	0.39**
	PN	0.24*	0.22*	0.15	0.20	−0.00	0.26*
	PA	0.33**	0.33**	0.36**	0.32**	−0.13	0.41**
	SA	0.16	0.03	0.17	0.23*	−0.07	0.16
	CTQS	0.41**	0.31**	0.34**	0.35**	−0.01	0.42**
War and PTSD *n*=56							
	EA	0.04	−0.13	0.15	0.09	0.14	0.07
	EN	−0.01	−0.09	0.17	0.24	0.26*	0.10
	PN	−0.01	−0.09	0.18	0.11	0.06	0.05
	PA	0.02	0.25	0.34**	0.02	−0.04	0.19
	SA	0.04	−0.06	−0.12	−0.18	−0.10	−0.11
	CTQS	−0.02	−0.11	0.21	0.19	0.20	0.10
No war *n*=41							
	EA	−0.12	−0.14	0.04	−0.06	0.19	−0.14
	EN	−0.17	0.03	−0.12	0.10	0.25	−0.05
	PN	−0.05	−0.13	0.14	0.06	0.08	−0.06
	PA	0.06	0.09	0.18	0.01	−0.09	0.12
	SA	−0.17	−0.05	−0.17	−0.09	−0.04	−0.19
	CTQS	−0.15	−0.03	−0.01	0.05	0.20	−0.09
All *n*=203							
	EA	0.15*	0.16*	0.13	0.17*	0.07	0.18*
	EN	0.00	0.15*	0.04	0.24**	−0.01	0.12
	PN	0.15*	0.20**	0.18*	0.32***	0.05	0.24**
	PA	0.15*	0.24**	0.16*	0.15*	0.04	0.25***
	SA	0.06	0.18*	0.05	−0.09	−0.08	0.18
	CTQS	0.12	0.23***	0.15*	0.29**	0.16	0.23**

*Note*: Correlations are significant

* *p*<0.05

** *p*<0.01

*** *p*<0.001, two tailed test.

Regression analysis was used to test whether a father's CM predicted the offspring's CM. Results indicated that father CM explained 6% of variance in offspring CM in the overall sample [*R* ^2^=0.06, *F*(1, 186) = 11.04, *p*<0.001]. Sixteen percent of offspring CM variance was explained by father CM in the W group [*R* ^2^=0.16, *F*(1,89) = 16.93, *p <* 0.001]. However, father CM did not significantly predict children's CM in the WP and NW groups.

A factorial MANCOVA was applied to examine differences in offspring CM subtypes depending on father's war status and offspring gender, while controlling for father CM exposure. Significant effects were detected for father's war-related status [Pillai's trace = 0.116, *F*(10, 354) = 2.19, *p*=0.018, and partial *η* ^2^=0.058], and for gender [Pillai's trace = 0.090, *F*(5, 177) = 3.52, *p*=0.005, and partial *η* ^2^=0.09] when controlling for father CM subtypes. A significant covariate effect was detected for father's physical neglect [Pillai's trace = 0.062, *F*(5, 176) = 2.11, *p*=0.044, and partial *η* ^2^=0.034]. Interaction between father group and gender was not significant [Pillai's trace = 0.030, *F*(10, 356) = 0.538, *p =* 0.863]. Between-subject analysis revealed significant mean differences in emotional neglect and physical neglect of children. Larger scores for emotional and physical neglect were found in children of war veterans with PTSD, as confirmed by Tukey post-hoc mean comparisons (see Table 2). Gender effects were significant only for emotional abuse [*F*(1, 188) = 4.651, *p*=0.032], with higher scores for women (*M*=7.57, *SD*=3.05 versus *M*=6.81, *SD*=2.25 for men).

**Fig. 1:**
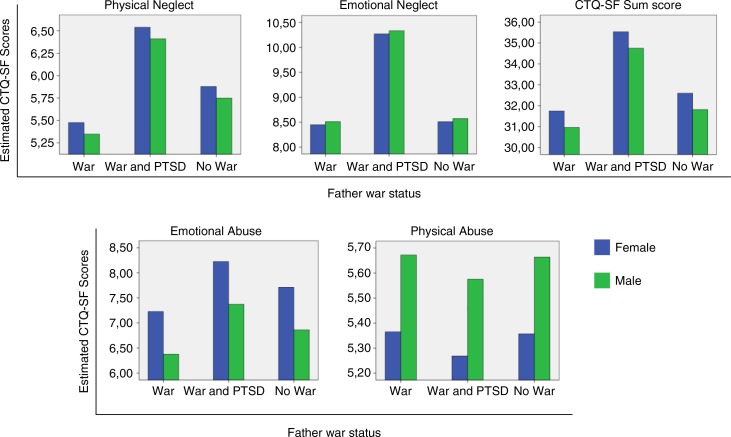
Estimated marginal means of adult self-reported childhood maltreatment, predicted by father's war status and gender, when controlling for childhood father's physical neglect exposure (covariate variable mean = 8.128), performed using multivariate analysis of covariance. Emotional neglect, physical neglect, and CTQ-SF sum scores are higher in the offspring of war veterans with PTSD. Emotional and physical abuse significantly differed by gender, but not by father's war status.

A second MANCOVA was performed including only the father's physical neglect as a covariate because it was the only significant covariate. Results for offspring CM estimates are summarized in Table 4. As shown by partial eta squared values, included variables predicted roughly 9% of variance of the offspring CM subtypes except for sexual abuse. Children emotional abuse was predicted by gender (*η* ^2^=0.025) and by father's physical neglect (*η* ^2^=0.038). Emotional neglect was predicted by father's war status (*η* ^2^=0.061) and by father's physical neglect (*η* ^2^=0.031). Offspring physical neglect was predicted by father's war status (*η* ^2^=0.083) and offspring physical abuse was only predicted by father's physical neglect (*η* ^2^=0.071). Figure 1 summarizes the estimated marginal means for the offspring CM subtypes and CTQS by gender and father's war status group, controlling for father's physical neglect.

**Table 4:** Multivariate analysis of covariance predicting offspring childhood maltreatment subtypes by gender and father's war status when controlling for father's childhood physical neglect

	Predictors								
	
	Model			Gender		Father's war S		Father PN	
				
Dependent variables	*F*	*p*	*R* ^2^	*η* ^2^	*p*	*η* ^2^	*p*	*η* ^2^	*p*
Emotional abuse	4.50	0.002	0.090*	0.025*	0.032	0.026	0.093	0.038*	0.008
Emotional neglect	4.85	0.001	0.096*	0.001	0.896	0.061*	0.003	0.031*	0.017
Physical neglect	5.05	0.001	0.099*	0.002	0.568	0.083*	0.000	0.015	0.098
Physical abuse	4.13	0.003	0.083*	0.015	0.101	0.001	0.897	0.071*	0.000
Sexual abuse	1.45	0.220	0.038	0.023*	0.038	0.004	0.688	0.00	0.745

* *Note*: Significant effect sizes. Father's war S, Father's war status; Father PN, father's childhood physical neglect. *n*=203.

## Discussion

Our study analyzed whether a father's war exposure and war-related PTSD diagnosis influenced maltreatment of his offspring. No difference was found between the extent of either physical or emotional neglect reported by children of Portuguese war exposed fathers and that reported by children whose fathers had not been exposed to the colonial war. However, an increased incidence of physical and emotional neglect was reported by those whose fathers had been war-exposed and had developed PTSD. This outcome contrasts with the results of research by Rentz et al. (2007) who found increased rates of CM among military families with deployed parents, independent of father's PTSD. Our results are in line with findings by Yehuda et al. (2001) and by Dekel and Goldblatt (2008) referencing the positive relationship between parental PTSD and CM; they also add strength to the hypothesis that CM can be a potential way of transmitting parental PTSD to the offspring, as suggested by Leen-Feldner et al. (2013). However, other mental disorders might have interfered with paternal parenting skills, including personality disorders, which are often concomitant clinical conditions.

Self-reported neglect experiences of children of war veterans with PTSD could have been related to fathers’ absence during war deployment. Nevertheless, children of W fathers who did not suffer PTSD did not show higher scores of physical or emotional neglect. This suggests that neglect is a consequence of the father's PTSD diagnosis. Because we did not control for the time frame of fathers’ physical absence during the children's upbringing, we cannot disentangle the effects of the fathers’ absence from the fathers’ psychopathology.

Considering that about 11% of Portuguese war-exposed men developed PTSD as a war exposure sequel (Albuquerque et al., 2003) and that 1 million men fought in the long and gruesome colonial war, with potentially deleterious effects on their children from CM, our results are relevant.

### CM in fathers

CM differed across the groups of fathers. WP fathers reported more CM, specifically with regard to emotional and physical abuse. Nevertheless, WP fathers and NW fathers registered similar emotional abuse during childhood. NW fathers could have been exposed to familial or social instability during their childhood and adolescence because most of them had to leave the country to escape war exposure. This fact might explain the comparable scores for emotional abuse among NW and WP father groups.

As indicated by previous studies (Berntsen et al., 2012; Bremner et al., 1993; Lanius et al., 2010), early life adversity plays an important role in the development of psychopathology, including PTSD. As suggested by Rademaker, Vermetten, Geuze, Muilwijk, and Kleber (2008), probable pathways linking CM to adult PTSD may rely (among other causes) on the development of dysfunctional personality traits in relation to self-directedness and cooperativeness, which are important traits for parenting behavior.

### Is there intergenerational transmission of CM?

Father CM explained 16% of the variance in the offspring CM in the group of W fathers. However, father–child correlations across groups differed, suggesting the existence of different patterns of CM transmission, depending on fathers’ war status. The main significant correlations appeared in the war-exposed group. Father CM exposure did not predict offspring CM in the war-exposed with PTSD or in the non-war-exposed groups. These results suggest that war exposure might have a mediating effect on the intergenerational transmission of CM. Nevertheless, group sizes varied; thus, results might have been influenced by this fact.

Correlations between father and child CM were not significant in the non-war-exposed group. Because better education levels were found in this group of fathers, education was perhaps a protecting factor against CM intergenerational transmission.

Children of WP fathers revealed similar levels of emotional abuse, physical abuse, and sexual abuse, when compared with W and NW groups. Only emotional neglect and physical neglect were larger. Because WP fathers experienced higher emotional and physical abuse during their infancy, and recognizing that war exposure might increase the risk for CM transmission, higher scores on these subtypes were expected in their children. Studies by Yehuda et al. (2001) of Holocaust families and by Ridier and Elbert (2013) of post-genocide victims from Rwanda also found higher scores for physical and emotional abuse in the offspring of parents with PTSD. However, our results did not provide full support for this association. Although our findings suggest that a father's PTSD diagnosis might be a probable cause of offspring neglect, WP fathers did not transmit childhood abusive experiences to their children. Fathers’ PTSD diagnosis may have had a defensive effect against abuse transmission, as was also found by Pears and Capaldi (2001).

Offspring CM was predicted by father's war status, father's physical neglect, and gender. Female gender is associated with an increased risk for emotional abuse exposure, after controlling for father's physical neglect. Despite the fact that effect sizes were modest, our results unveil the hypothesis for heterogeneous patterns of intergenerational transmission of CM: parents exposed to neglect may transmit abuse, while parents exposed to abuse may transmit neglect.

Taking into account these findings, PTSD of Portuguese war veterans may have led to emotional dysregulation (Lanius et al., 2010), emotional detachment (Ruscio, Weathers, King, & King, 2002), and avoidance (Dekel & Monson, 2010) or related consequences, resulting in neglect of their offspring. Instead of transmitting their own childhood abuse experiences, WP fathers neglected their offspring to some extent.

Considering that the WP fathers included in our study have been followed in psychiatric consultations for more than 10 years, we infer that the severity of PTSD can have profoundly impacted their familial interactions, as suggested by Pears and Capaldi (2001). Because neglect is a form of care omission, our results add strength to the hypothesis of the deleterious effects of PTSD on parenting skills, serving as a probable barrier to father–child interaction.

Specific cultural and societal background characteristics may need to be examined to explain the increased neglect exposure among children of Portuguese fathers with war-related PTSD (Kleber, Figley, & Gersons, 1995). The perceived senselessness of Portuguese veterans’ war participation (Sales, 2006; Schok, Kleber, Elands, & Weerts, 2008), and the fact that war scenarios are largely unknown by society in general (Ribeiro et al., 2011), might have acted as barriers in the psychological recovery of Portuguese war veterans who have undergone war trauma. War injuries might have become socially invisible and unrecognized by the veteran′s family, increasing their hurtful potential for war veterans. The silence that characterized the post-war period in Portugal may have contributed to strong levels of avoidance of their wartime experiences. It is likely that war veterans with PTSD became even more distant from their spouses and children because they found it impossible to disclose their own past experiences.

### Limitations and strengths

A limitation of this study is that although the groups we examined were equivalent in terms of their sociodemographic characteristics, we are not certain that the samples were representative of the Portuguese population, due to the use of the snowball technique.

Because the group of participating children were all adults at the time of assessment, actual father PTSD measures may not elucidate the effects of earlier symptomatology on offspring CM. Data about fathers’ PTSD symptoms during their offspring's upbringing were not available, and knowledge about comorbidity is scarce. In addition, the fact that war veterans did not receive psychiatric treatment does not ensure they did not have PTSD symptoms during their children's development. Another source of information is missing in our study—we did not analyze mental health and CM with regard to mothers.

Assessment of CM was based on retrospective self-reported measures, which can fail to detect CM exposure because of denial mechanisms or memory degradation. In addition, the Portuguese version of the CTQ-SF used in this study presented low internal consistency in the subscale of physical neglect, and the CTQ-SF does not allow the identification of the perpetrators, leaving some ambiguity in the interpretation of the causes for the increased levels of neglect in the offspring of war veterans with PTSD.

Although data were collected with a self-reported retrospective measure, the instrument is widely used (Thornberry et al., 2012) and was validated for the Portuguese population (Dias et al., 2013). Another strength of the study is that we assessed fathers and children, reducing possible biases related to the instrument itself. Gilbert et al. (2009) stated that official reports account for less than one-tenth of actual CM prevalence rates, making self-report assessment more appropriate for exploring the broader spectrum of CM exposure. Using community samples and a control group have strengthened our findings because confounding variables from at-risk or clinical populations could thus be reduced. Our groups of children and fathers belonged to close generations. Thus, the effects of economical and sociocultural factors on the CM results were minimized.

### Future directions

Our work emphasizes the potential risk of neglect exposure for children of war veterans with PTSD. Taking into account the relations found between father abuse and child neglect, the hypothesis of heterogeneous intergenerational transmission of CM should be considered in future research work. Nevertheless, our results need to be replicated using a prospective methodology and including representative community populations from other cultural contexts, in order to avoid sample selection biases. The group of fathers with PTSD diagnosis had also been exposed to increased levels of childhood abuse. Further work is required to differentiate between effects of fathers’ PTSD symptoms and fathers’ childhood abuse exposure on the levels of offspring neglect.

Although children of war veterans with PTSD have reported higher physical and emotional neglect, this does not imply maladaptive functioning. Thus, data on children's adjustment should be addressed. Additionally, data on mental health and CM exposure concerning both parents would allow a better understanding of the contribution of fathers’ PTSD for neglect of children. The independent effects of parental physical absence should be sorted out from the effects of PTSD or other related variables.

## Conclusions

The adult offspring of Portuguese war veterans with PTSD reported higher levels of CM, in terms of emotional and physical neglect. War veterans with PTSD also reported having experienced more emotional and physical abuse in their own childhood. However, they were less likely to transmit their childhood abusive experiences to their children, compared with war veterans without PTSD. Thus, a father's war-related PTSD might be a risk factor for offspring neglect but potentially a protective one for the father–child abuse transmission. War exposed fathers without PTSD did transmit their own CM experiences more often. Therefore, father's war exposure and father's war PTSD appeared as important variables in the study of the intergenerational transmission of CM.
